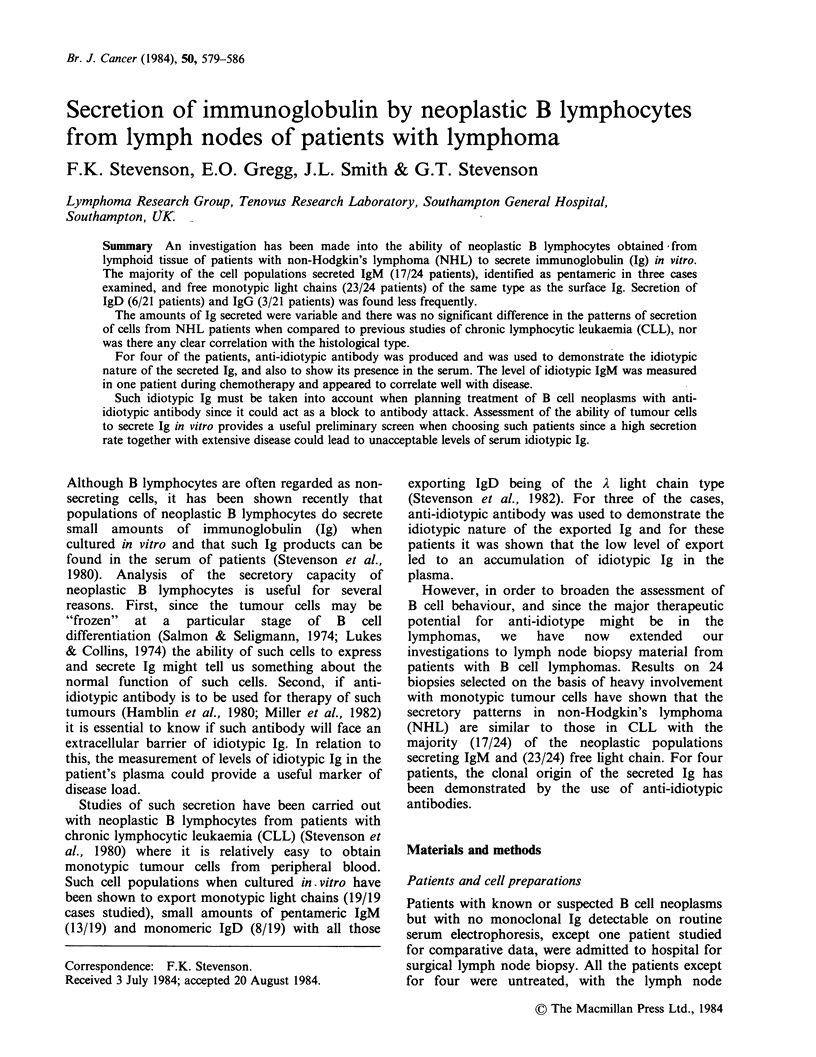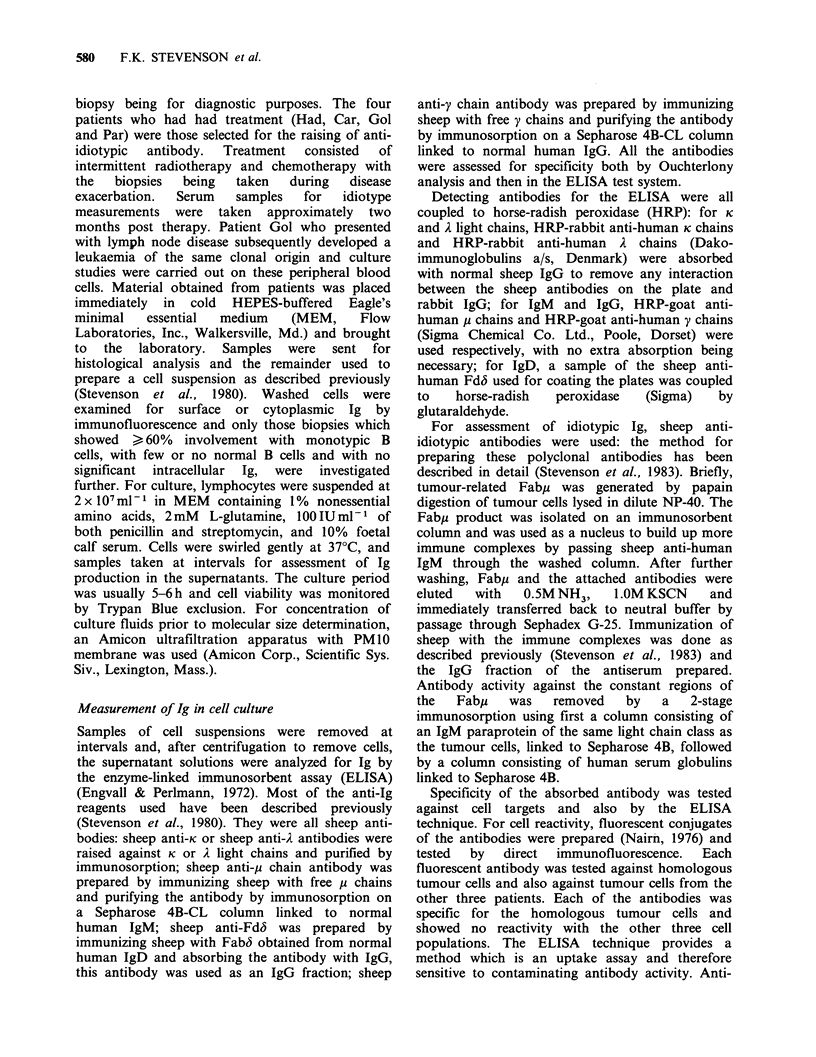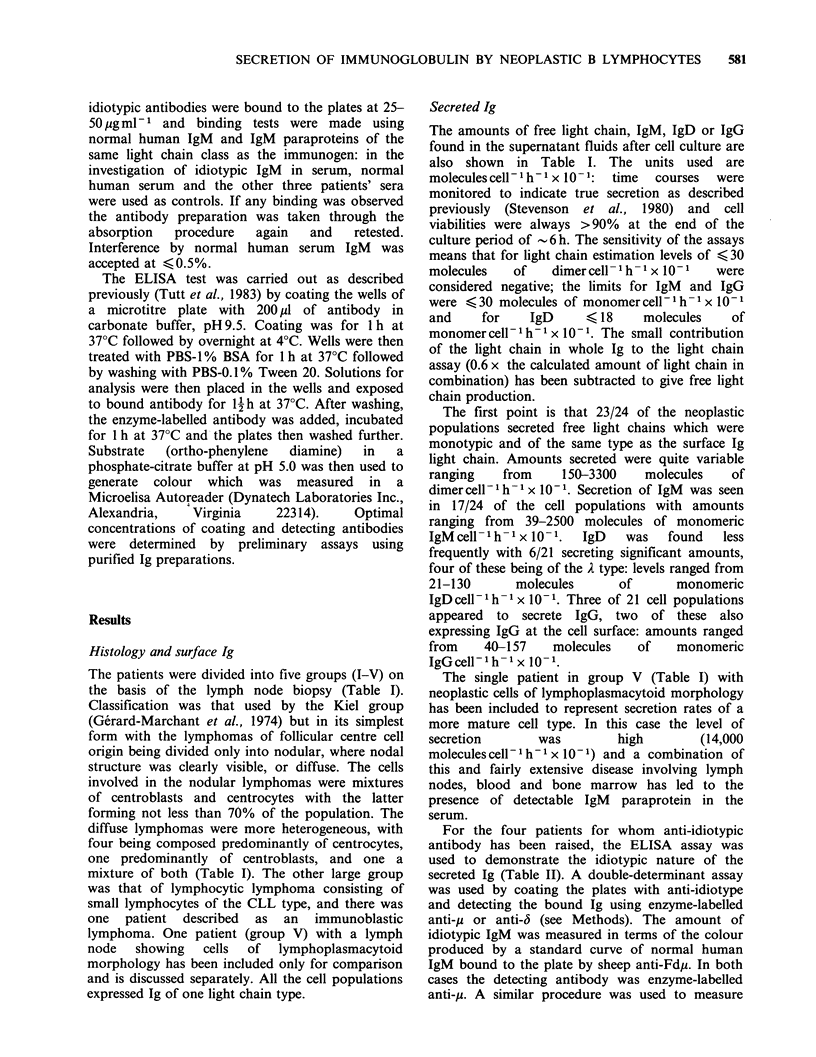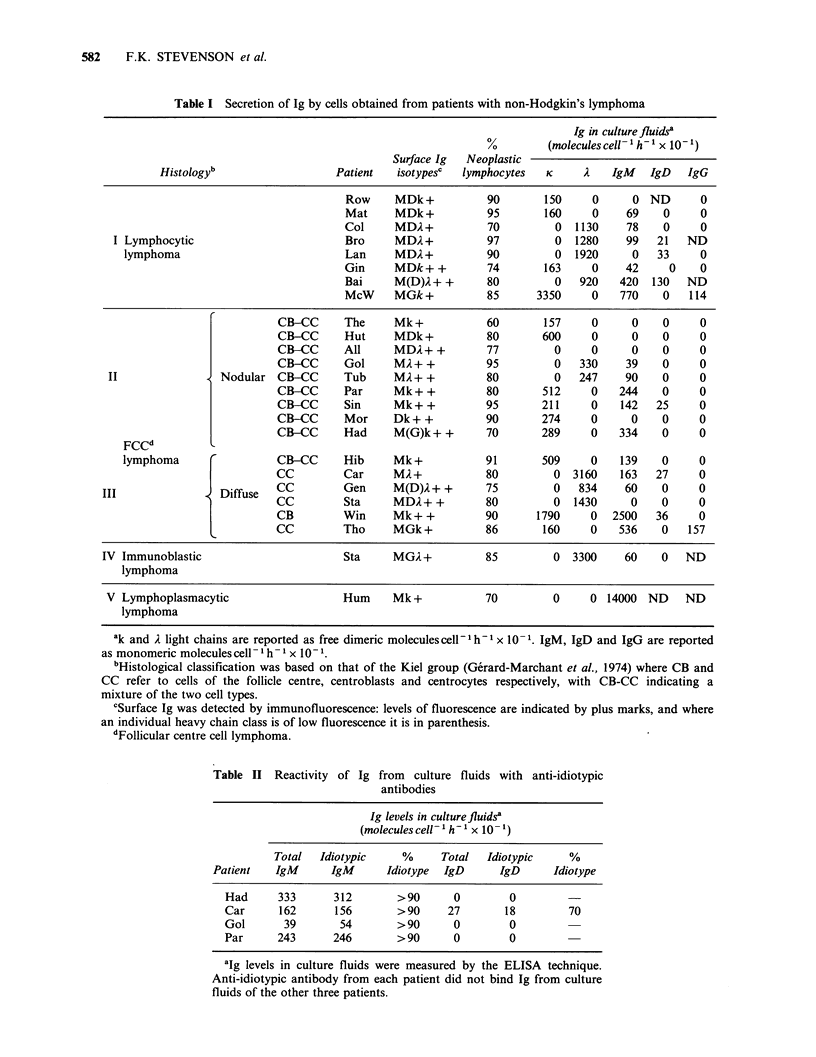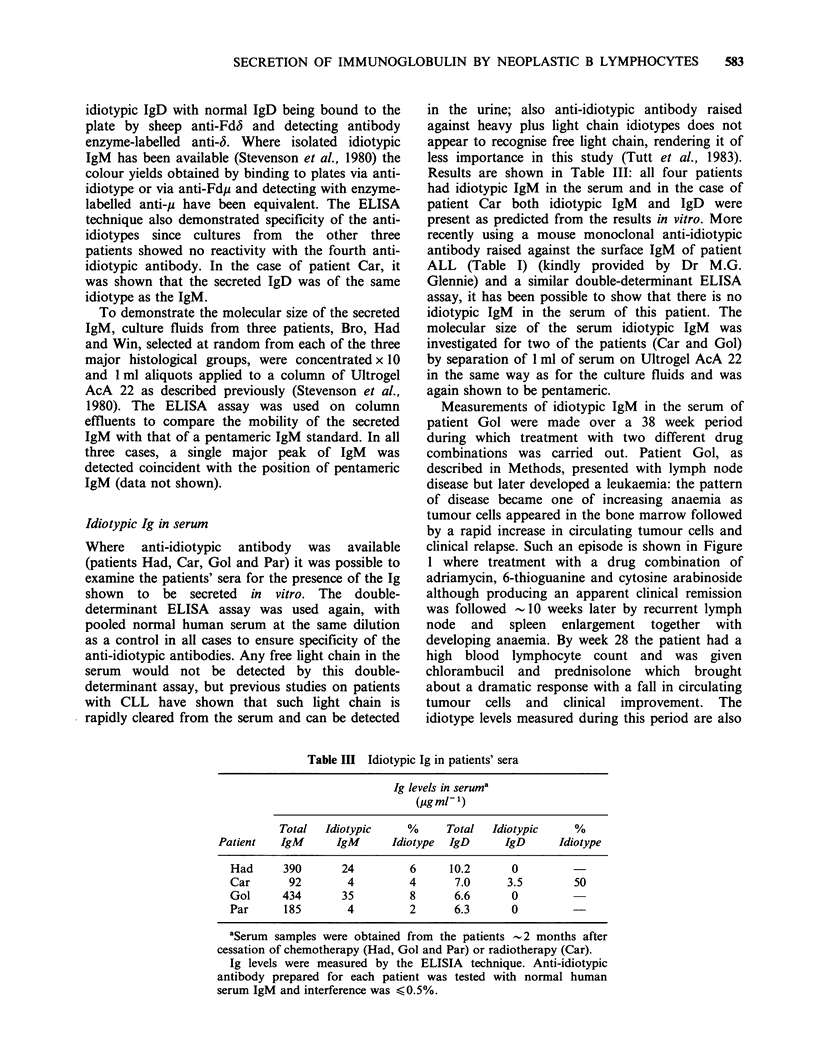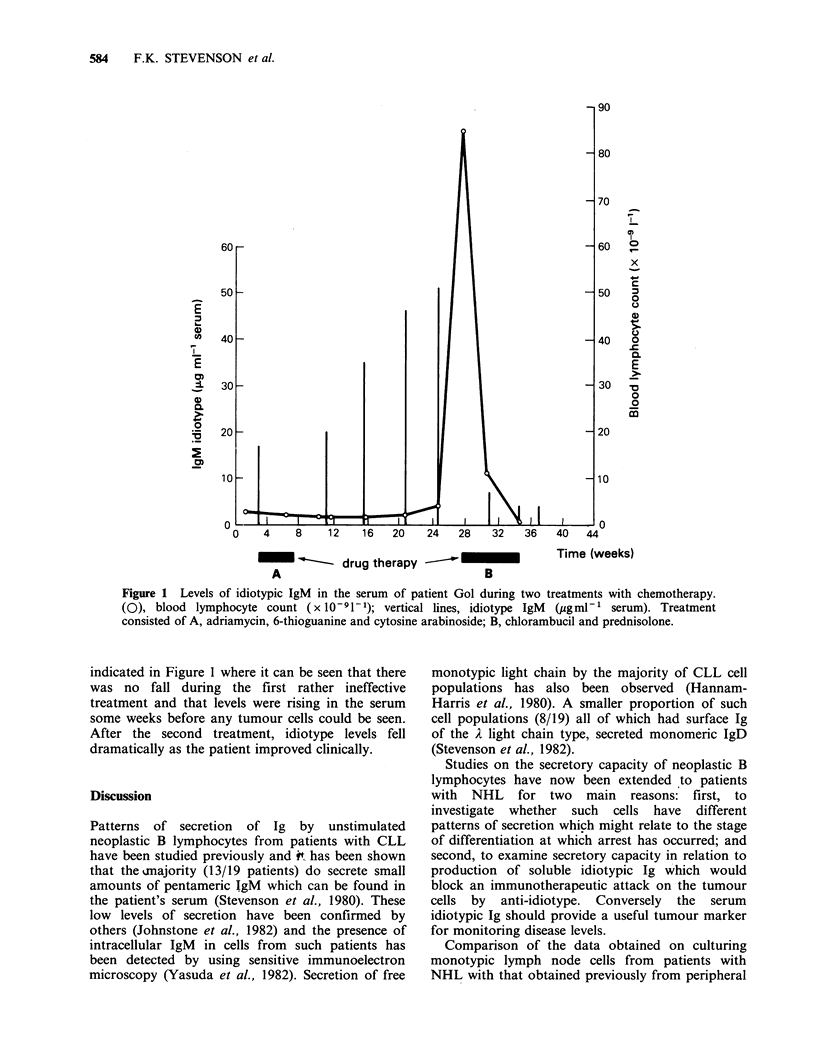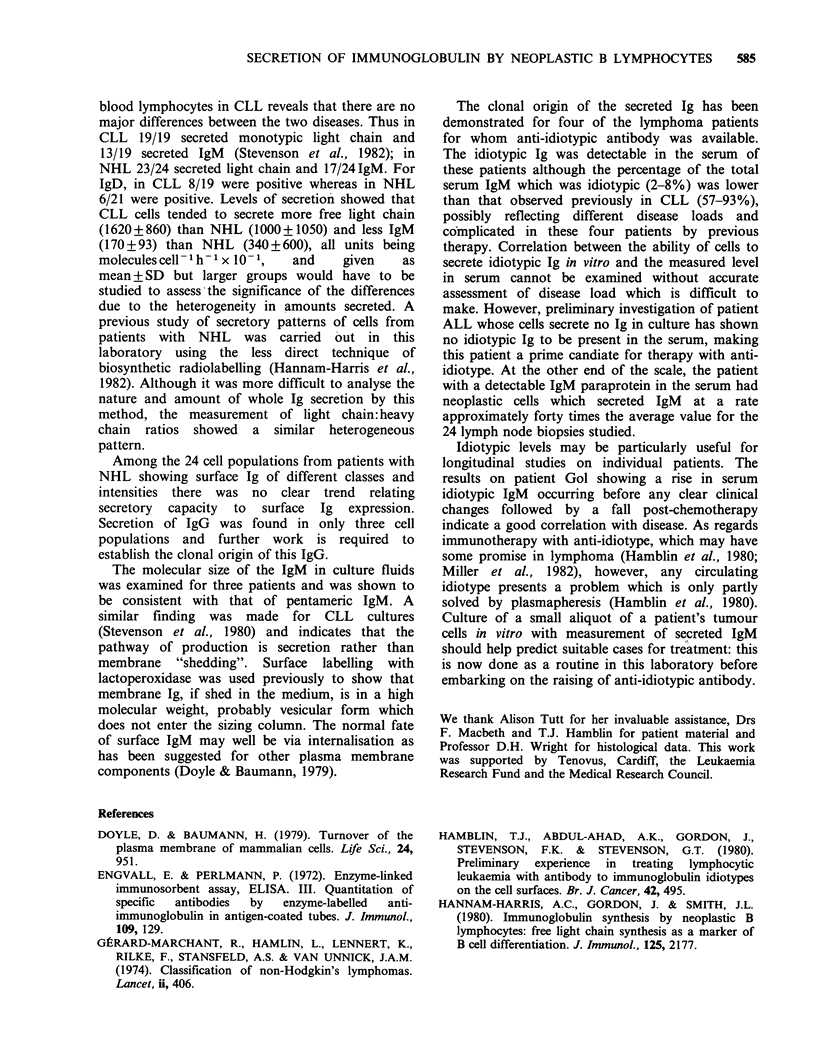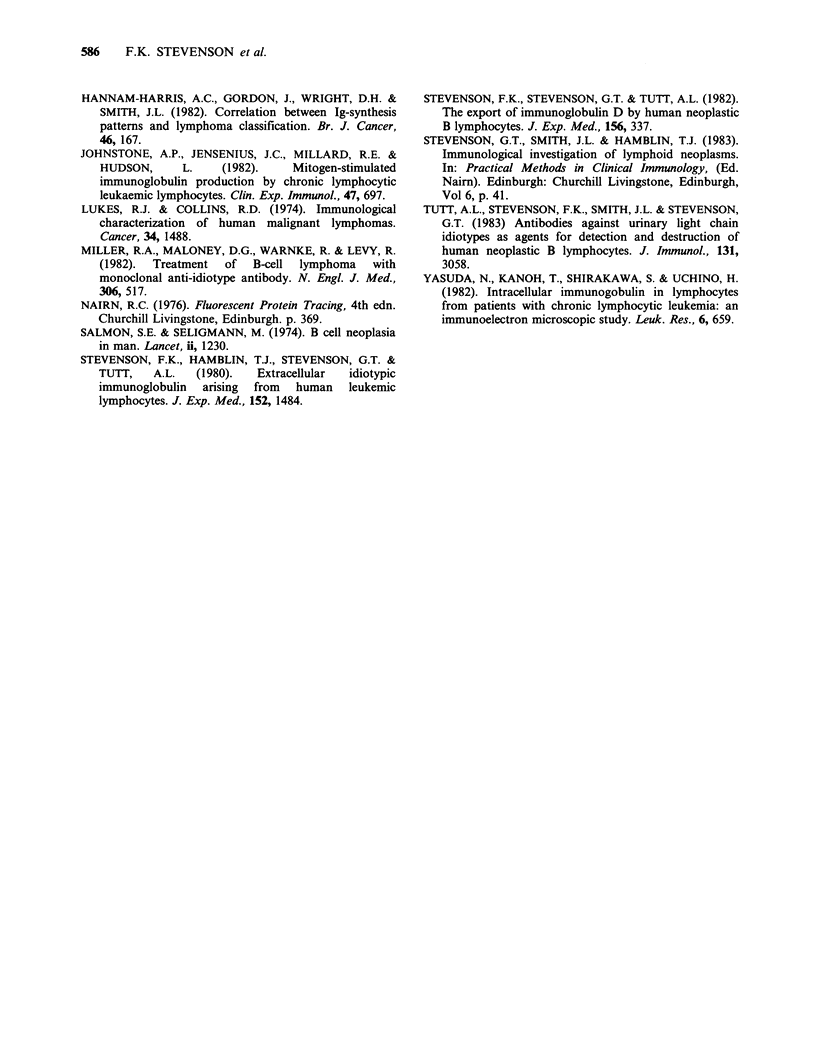# Secretion of immunoglobulin by neoplastic B lymphocytes from lymph nodes of patients with lymphoma.

**DOI:** 10.1038/bjc.1984.224

**Published:** 1984-11

**Authors:** F. K. Stevenson, E. O. Gregg, J. L. Smith, G. T. Stevenson

## Abstract

An investigation has been made into the ability of neoplastic B lymphocytes obtained from lymphoid tissue of patients with non-Hodgkin's lymphoma (NHL) to secrete immunoglobulin (Ig) in vitro. The majority of the cell populations secreted IgM (17/24 patients), identified as pentameric in three cases examined, and free monotypic light chains (23/24 patients) of the same type as the surface Ig. Secretion of IgD (6/21 patients) and IgG (3/21 patients) was found less frequently. The amounts of Ig secreted were variable and there was no significant difference in the patterns of secretion of cells from NHL patients when compared to previous studies of chronic lymphocytic leukaemia (CLL), nor was there any clear correlation with the histological type. For four of the patients, anti-idiotypic antibody was produced and was used to demonstrate the idiotypic nature of the secreted Ig, and also to show its presence in the serum. The level of idiotypic IgM was measured in one patient during chemotherapy and appeared to correlate well with disease. Such idiotypic Ig must be taken into account when planning treatment of B cell neoplasms with antiidiotypic antibody since it could act as a block to antibody attack. Assessment of the ability of tumour cells to secrete Ig in vitro provides a useful preliminary screen when choosing such patients since a high secretion rate together with extensive disease could lead to unacceptable levels of serum idiotypic Ig.


*Br. J. Cancer* (1984), **50**, 579–586

### Secretion of immunoglobulin by neoplastic B lymphocytes from lymph nodes of patients with lymphoma

F.K. Stevenson, E.O. Gregg, J.L. Smith & G.T. Stevenson

*Lymphoma Research Group, Tenovus Research Laboratory, Southampton General Hospital, Southampton, UK.*

**Summary** An investigation has been made into the ability of neoplastic B lymphocytes obtained from lymphoid tissue of patients with non-Hodgkin's lymphoma (NHL) to secrete immunoglobulin (Ig) *in vitro*. The majority of the cell populations secreted IgM (17/24 patients), identified as pentameric in three cases examined, and free monotypic light chains (23/24 patients) of the same type as the surface Ig. Secretion of IgD (6/21 patients) and IgG (3/21 patients) was found less frequently.

The amounts of Ig secreted were variable and there was no significant difference in the patterns of secretion of cells from NHL patients when compared to previous studies of chronic lymphocytic leukaemia (CLL), nor was there any clear correlation with the histological type.

For four of the patients, anti-idiotypic antibody was produced and was used to demonstrate the idiotypic nature of the secreted Ig, and also to show its presence in the serum. The level of idiotypic IgM was measured in one patient during chemotherapy and appeared to correlate well with disease.

Such idiotypic Ig must be taken into account when planning treatment of B cell neoplasms with anti-idiotypic antibody since it could act as a block to antibody attack. Assessment of the ability of tumour cells to secrete Ig *in vitro* provides a useful preliminary screen when choosing such patients since a high secretion rate together with extensive disease could lead to unacceptable levels of serum idiotypic Ig.

Although B lymphocytes are often regarded as non-secreting cells, it has been shown recently that populations of neoplastic B lymphocytes do secrete small amounts of immunoglobulin (Ig) when cultured *in vitro* and that such Ig products can be found in the serum of patients (Stevenson *et al.*, 1980). Analysis of the secretory capacity of neoplastic B lymphocytes is useful for several reasons. First, since the tumour cells may be "frozen" at a particular stage of B cell differentiation (Salmon & Seligmann, 1974; Lukes & Collins, 1974) the ability of such cells to express and secrete Ig might tell us something about the normal function of such cells. Second, if anti-idiotypic antibody is to be used for therapy of such tumours (Hamblin *et al.*, 1980; Miller *et al.*, 1982) it is essential to know if such antibody will face an extracellular barrier of idiotypic Ig. In relation to this, the measurement of levels of idiotypic Ig in the patient's plasma could provide a useful marker of disease load.

Studies of such secretion have been carried out with neoplastic B lymphocytes from patients with chronic lymphocytic leukaemia (CLL) (Stevenson *et al.*, 1980) where it is relatively easy to obtain monotypic tumour cells from peripheral blood. Such cell populations when cultured *in vitro* have been shown to export monotypic light chains (19/19 cases studied), small amounts of pentameric IgM (13/19) and monomeric IgD (8/19) with all those exporting IgD being of the $\lambda$ light chain type (Stevenson *et al.*, 1982). For three of the cases, anti-idiotypic antibody was used to demonstrate the idiotypic nature of the exported Ig and for these patients it was shown that the low level of export led to an accumulation of idiotypic Ig in the plasma.

However, in order to broaden the assessment of B cell behaviour, and since the major therapeutic potential for anti-idiotype might be in the lymphomas, we have now extended our investigations to lymph node biopsy material from patients with B cell lymphomas. Results on 24 biopsies selected on the basis of heavy involvement with monotypic tumour cells have shown that the secretory patterns in non-Hodgkin's lymphoma (NHL) are similar to those in CLL with the majority (17/24) of the neoplastic populations secreting IgM and (23/24) free light chain. For four patients, the clonal origin of the secreted Ig has been demonstrated by the use of anti-idiotypic antibodies.

### Materials and methods

### *Patients and cell preparations*

Patients with known or suspected B cell neoplasms but with no monoclonal Ig detectable on routine serum electrophoresis, except one patient studied for comparative data, were admitted to hospital for surgical lymph node biopsy. All the patients except for four were untreated, with the lymph node

Correspondence: F.K. Stevenson.
Received 3 July 1984; accepted 20 August 1984.

© The Macmillan Press Ltd., 1984

biopsy being for diagnostic purposes. The four patients who had had treatment (Had, Car, Gol and Par) were those selected for the raising of anti-idiotypic antibody. Treatment consisted of intermittent radiotherapy and chemotherapy with the biopsies being taken during disease exacerbation. Serum samples for idiotype measurements were taken approximately two months post therapy. Patient Gol who presented with lymph node disease subsequently developed a leukaemia of the same clonal origin and culture studies were carried out on these peripheral blood cells. Material obtained from patients was placed immediately in cold HEPES-buffered Eagle's minimal essential medium (MEM, Flow Laboratories, Inc., Walkersville, Md.) and brought to the laboratory. Samples were sent for histological analysis and the remainder used to prepare a cell suspension as described previously (Stevenson *et al.,* 1980). Washed cells were examined for surface or cytoplasmic Ig by immunofluorescence and only those biopsies which showed $\geqslant 60\%$ involvement with monotypic B cells, with few or no normal B cells and with no significant intracellular Ig, were investigated further. For culture, lymphocytes were suspended at $2 \times 10^7 ml^{-1}$ in MEM containing 1% nonessential amino acids, 2 mM L-glutamine, 100 IU $ml^{-1}$ of both penicillin and streptomycin, and 10% foetal calf serum. Cells were swirled gently at 37°C, and samples taken at intervals for assessment of Ig production in the supernatants. The culture period was usually 5–6 h and cell viability was monitored by Trypan Blue exclusion. For concentration of culture fluids prior to molecular size determination, an Amicon ultrafiltration apparatus with PM10 membrane was used (Amicon Corp., Scientific Sys. Siv., Lexington, Mass.).

*Measurement of Ig in cell culture*

Samples of cell suspensions were removed at intervals and, after centrifugation to remove cells, the supernatant solutions were analyzed for Ig by the enzyme-linked immunosorbent assay (ELISA) (Engvall & Perlmann, 1972). Most of the anti-Ig reagents used have been described previously (Stevenson *et al.*, 1980). They were all sheep antibodies: sheep anti-$\kappa$ or sheep anti-$\lambda$ antibodies were raised against $\kappa$ or $\lambda$ light chains and purified by immunosorption; sheep anti-$\mu$ chain antibody was prepared by immunizing sheep with free $\mu$ chains and purifying the antibody by immunosorption on a Sepharose 4B-CL column linked to normal human IgM; sheep anti-Fd$\delta$ was prepared by immunizing sheep with Fab$\delta$ obtained from normal human IgD and absorbing the antibody with IgG, this antibody was used as an IgG fraction; sheep anti-$\gamma$ chain antibody was prepared by immunizing sheep with free $\gamma$ chains and purifying the antibody by immunosorption on a Sepharose 4B-CL column linked to normal human IgG. All the antibodies were assessed for specificity both by Ouchterlony analysis and then in the ELISA test system.

Detecting antibodies for the ELISA were all coupled to horse-radish peroxidase (HRP): for $\kappa$ and $\lambda$ light chains, HRP-rabbit anti-human $\kappa$ chains and HRP-rabbit anti-human $\lambda$ chains (Dako-immunoglobulins a/s, Denmark) were absorbed with normal sheep IgG to remove any interaction between the sheep antibodies on the plate and rabbit IgG; for IgM and IgG, HRP-goat anti-human $\mu$ chains and HRP-goat anti-human $\gamma$ chains (Sigma Chemical Co. Ltd., Poole, Dorset) were used respectively, with no extra absorption being necessary; for IgD, a sample of the sheep anti-human Fd$\delta$ used for coating the plates was coupled to horse-radish peroxidase (Sigma) by glutaraldehyde.

For assessment of idiotypic Ig, sheep anti-idiotypic antibodies were used: the method for preparing these polyclonal antibodies has been described in detail (Stevenson *et al.*, 1983). Briefly, tumour-related Fab$\mu$ was generated by papain digestion of tumour cells lysed in dilute NP-40. The Fab$\mu$ product was isolated on an immunosorbent column and was used as a nucleus to build up more immune complexes by passing sheep anti-human IgM through the washed column. After further washing, Fab$\mu$ and the attached antibodies were eluted with 0.5M $NH_3$, 1.0M KSCN and immediately transferred back to neutral buffer by passage through Sephadex G-25. Immunization of sheep with the immune complexes was done as described previously (Stevenson *et al.,* 1983) and the IgG fraction of the antiserum prepared. Antibody activity against the constant regions of the Fab$\mu$ was removed by a 2-stage immunosorption using first a column consisting of an IgM paraprotein of the same light chain class as the tumour cells, linked to Sepharose 4B, followed by a column consisting of human serum globulins linked to Sepharose 4B.

Specificity of the absorbed antibody was tested against cell targets and also by the ELISA technique. For cell reactivity, fluorescent conjugates of the antibodies were prepared (Nairn, 1976) and tested by direct immunofluorescence. Each fluorescent antibody was tested against homologous tumour cells and also against tumour cells from the other three patients. Each of the antibodies was specific for the homologous tumour cells and showed no reactivity with the other three cell populations. The ELISA technique provides a method which is an uptake assay and therefore sensitive to contaminating antibody activity. Antiidiotypic antibodies were bound to the plates at 25–50 $\mu g\,ml^{-1}$ and binding tests were made using normal human IgM and IgM paraproteins of the same light chain class as the immunogen: in the investigation of idiotypic IgM in serum, normal human serum and the other three patients' sera were used as controls. If any binding was observed the antibody preparation was taken through the absorption procedure again and retested. Interference by normal human serum IgM was accepted at $\leqslant 0.5\%$.

The ELISA test was carried out as described previously (Tutt *et al.*, 1983) by coating the wells of a microtitre plate with 200 $\mu l$ of antibody in carbonate buffer, pH 9.5. Coating was for 1 h at 37°C followed by overnight at 4°C. Wells were then treated with PBS-1% BSA for 1 h at 37°C followed by washing with PBS-0.1% Tween 20. Solutions for analysis were then placed in the wells and exposed to bound antibody for $1\frac{1}{2}$ h at 37°C. After washing, the enzyme-labelled antibody was added, incubated for 1 h at 37°C and the plates then washed further. Substrate (ortho-phenylene diamine) in a phosphate-citrate buffer at pH 5.0 was then used to generate colour which was measured in a Microelisa Autoreader (Dynatech Laboratories Inc., Alexandria, Virginia 22314). Optimal concentrations of coating and detecting antibodies were determined by preliminary assays using purified Ig preparations.

### Results

### *Histology and surface Ig*

The patients were divided into five groups (I–V) on the basis of the lymph node biopsy (Table I). Classification was that used by the Kiel group (Gérard-Marchant *et al.*, 1974) but in its simplest form with the lymphomas of follicular centre cell origin being divided only into nodular, where nodal structure was clearly visible, or diffuse. The cells involved in the nodular lymphomas were mixtures of centroblasts and centrocytes with the latter forming not less than 70% of the population. The diffuse lymphomas were more heterogeneous, with four being composed predominantly of centrocytes, one predominantly of centroblasts, and one a mixture of both (Table I). The other large group was that of lymphocytic lymphoma consisting of small lymphocytes of the CLL type, and there was one patient described as an immunoblastic lymphoma. One patient (group V) with a lymph node showing cells of lymphoplasmacytoid morphology has been included only for comparison and is discussed separately. All the cell populations expressed Ig of one light chain type.

### *Secreted Ig*

The amounts of free light chain, IgM, IgD or IgG found in the supernatant fluids after cell culture are also shown in Table I. The units used are molecules $cell^{-1}\,h^{-1} \times 10^{-1}$: time courses were monitored to indicate true secretion as described previously (Stevenson *et al.*, 1980) and cell viabilities were always $>90\%$ at the end of the culture period of $\sim 6$ h. The sensitivity of the assays means that for light chain estimation levels of $\leqslant 30$ molecules of dimer $cell^{-1}\,h^{-1} \times 10^{-1}$ were considered negative; the limits for IgM and IgG were $\leqslant 30$ molecules of monomer $cell^{-1}\,h^{-1} \times 10^{-1}$ and for IgD $\leqslant 18$ molecules of monomer $cell^{-1}\,h^{-1} \times 10^{-1}$. The small contribution of the light chain in whole Ig to the light chain assay ($0.6\times$ the calculated amount of light chain in combination) has been subtracted to give free light chain production.

The first point is that 23/24 of the neoplastic populations secreted free light chains which were monotypic and of the same type as the surface Ig light chain. Amounts secreted were quite variable ranging from 150–3300 molecules of dimer $cell^{-1}\,h^{-1} \times 10^{-1}$. Secretion of IgM was seen in 17/24 of the cell populations with amounts ranging from 39–2500 molecules of monomeric IgM $cell^{-1}\,h^{-1} \times 10^{-1}$. IgD was found less frequently with 6/21 secreting significant amounts, four of these being of the $\lambda$ type: levels ranged from 21–130 molecules of monomeric IgD $cell^{-1}\,h^{-1} \times 10^{-1}$. Three of 21 cell populations appeared to secrete IgG, two of these also expressing IgG at the cell surface: amounts ranged from 40–157 molecules of monomeric IgG $cell^{-1}\,h^{-1} \times 10^{-1}$.

The single patient in group V (Table I) with neoplastic cells of lymphoplasmacytoid morphology has been included to represent secretion rates of a more mature cell type. In this case the level of secretion was high (14,000 molecules $cell^{-1}\,h^{-1} \times 10^{-1}$) and a combination of this and fairly extensive disease involving lymph nodes, blood and bone marrow has led to the presence of detectable IgM paraprotein in the serum.

For the four patients for whom anti-idiotypic antibody has been raised, the ELISA assay was used to demonstrate the idiotypic nature of the secreted Ig (Table II). A double-determinant assay was used by coating the plates with anti-idiotype and detecting the bound Ig using enzyme-labelled anti-$\mu$ or anti-$\delta$ (see Methods). The amount of idiotypic IgM was measured in terms of the colour produced by a standard curve of normal human IgM bound to the plate by sheep anti-Fd$\mu$. In both cases the detecting antibody was enzyme-labelled anti-$\mu$. A similar procedure was used to measure

**Table I** Secretion of Ig by cells obtained from patients with non-Hodgkin's lymphoma

| *Histology*[b] | | | *Patient* | *Surface Ig isotypes*[c] | *% Neoplastic lymphocytes* | *Ig in culture fluids*[a] *(molecules cell$^{-1}$ h$^{-1}$ × 10$^{-1}$)* κ | λ | *IgM* | *IgD* | *IgG* |
|---|---|---|---|---|---|---|---|---|---|---|
| I Lymphocytic lymphoma | | | Row | MDk+ | 90 | 150 | 0 | 0 | ND | 0 |
| | | | Mat | MDk+ | 95 | 160 | 0 | 69 | 0 | 0 |
| | | | Col | MDλ+ | 70 | 0 | 1130 | 78 | 0 | 0 |
| | | | Bro | MDλ+ | 97 | 0 | 1280 | 99 | 21 | ND |
| | | | Lan | MDλ+ | 90 | 0 | 1920 | 0 | 33 | 0 |
| | | | Gin | MD*k*++ | 74 | 163 | 0 | 42 | 0 | 0 |
| | | | Bai | M(D)λ++ | 80 | 0 | 920 | 420 | 130 | ND |
| | | | McW | MG*k*+ | 85 | 3350 | 0 | 770 | 0 | 114 |
| II FCC[d] lymphoma | Nodular | CB–CC | The | Mk+ | 60 | 157 | 0 | 0 | 0 | 0 |
| | | CB–CC | Hut | MDk+ | 80 | 600 | 0 | 0 | 0 | 0 |
| | | CB–CC | All | MDλ++ | 77 | 0 | 0 | 0 | 0 | 0 |
| | | CB–CC | Gol | Mλ++ | 95 | 0 | 330 | 39 | 0 | 0 |
| | | CB–CC | Tub | Mλ++ | 80 | 0 | 247 | 90 | 0 | 0 |
| | | CB–CC | Par | Mk++ | 80 | 512 | 0 | 244 | 0 | 0 |
| | | CB–CC | Sin | Mk++ | 95 | 211 | 0 | 142 | 25 | 0 |
| | | CB–CC | Mor | Dk++ | 90 | 274 | 0 | 0 | 0 | 0 |
| | | CB–CC | Had | M(G)k++ | 70 | 289 | 0 | 334 | 0 | 0 |
| III FCC[d] lymphoma | Diffuse | CB–CC | Hib | Mk+ | 91 | 509 | 0 | 139 | 0 | 0 |
| | | CC | Car | Mλ+ | 80 | 0 | 3160 | 163 | 27 | 0 |
| | | CC | Gen | M(D)λ++ | 75 | 0 | 834 | 60 | 0 | 0 |
| | | CC | Sta | MDλ++ | 80 | 0 | 1430 | 0 | 0 | 0 |
| | | CB | Win | Mk++ | 90 | 1790 | 0 | 2500 | 36 | 0 |
| | | CC | Tho | MGk+ | 86 | 160 | 0 | 536 | 0 | 157 |
| IV Immunoblastic lymphoma | | | Sta | MGλ+ | 85 | 0 | 3300 | 60 | 0 | ND |
| V Lymphoplasmacytic lymphoma | | | Hum | Mk+ | 70 | 0 | 0 | 14000 | ND | ND |

[a]k and λ light chains are reported as free dimeric molecules cell$^{-1}$ h$^{-1}$ × 10$^{-1}$. IgM, IgD and IgG are reported as monomeric molecules cell$^{-1}$ h$^{-1}$ × 10$^{-1}$.
[b]Histological classification was based on that of the Kiel group (Gérard-Marchant *et al.*, 1974) where CB and CC refer to cells of the follicle centre, centroblasts and centrocytes respectively, with CB-CC indicating a mixture of the two cell types.
[c]Surface Ig was detected by immunofluorescence: levels of fluorescence are indicated by plus marks, and where an individual heavy chain class is of low fluorescence it is in parenthesis.
[d]Follicular centre cell lymphoma.

**Table II** Reactivity of Ig from culture fluids with anti-idiotypic antibodies

| *Patient* | *Ig levels in culture fluids*[a] *(molecules cell$^{-1}$ h$^{-1}$ × 10$^{-1}$)* *Total IgM* | *Idiotypic IgM* | *% Idiotype* | *Total IgD* | *Idiotypic IgD* | *% Idiotype* |
|---|---|---|---|---|---|---|
| Had | 333 | 312 | >90 | 0 | 0 | — |
| Car | 162 | 156 | >90 | 27 | 18 | 70 |
| Gol | 39 | 54 | >90 | 0 | 0 | — |
| Par | 243 | 246 | >90 | 0 | 0 | — |

[a]Ig levels in culture fluids were measured by the ELISA technique. Anti-idiotypic antibody from each patient did not bind Ig from culture fluids of the other three patients.

idiotypic IgD with normal IgD being bound to the plate by sheep anti-Fdδ and detecting antibody enzyme-labelled anti-δ. Where isolated idiotypic IgM has been available (Stevenson *et al.*, 1980) the colour yields obtained by binding to plates via anti-idiotype or via anti-Fdμ and detecting with enzyme-labelled anti-μ have been equivalent. The ELISA technique also demonstrated specificity of the anti-idiotypes since cultures from the other three patients showed no reactivity with the fourth anti-idiotypic antibody. In the case of patient Car, it was shown that the secreted IgD was of the same idiotype as the IgM.

To demonstrate the molecular size of the secreted IgM, culture fluids from three patients, Bro, Had and Win, selected at random from each of the three major histological groups, were concentrated × 10 and 1 ml aliquots applied to a column of Ultrogel AcA 22 as described previously (Stevenson *et al.*, 1980). The ELISA assay was used on column effluents to compare the mobility of the secreted IgM with that of a pentameric IgM standard. In all three cases, a single major peak of IgM was detected coincident with the position of pentameric IgM (data not shown).

### *Idiotypic Ig in serum*

Where anti-idiotypic antibody was available (patients Had, Car, Gol and Par) it was possible to examine the patients' sera for the presence of the Ig shown to be secreted *in vitro*. The double-determinant ELISA assay was used again, with pooled normal human serum at the same dilution as a control in all cases to ensure specificity of the anti-idiotypic antibodies. Any free light chain in the serum would not be detected by this double-determinant assay, but previous studies on patients with CLL have shown that such light chain is rapidly cleared from the serum and can be detected in the urine; also anti-idiotypic antibody raised against heavy plus light chain idiotypes does not appear to recognise free light chain, rendering it of less importance in this study (Tutt *et al.*, 1983). Results are shown in Table III: all four patients had idiotypic IgM in the serum and in the case of patient Car both idiotypic IgM and IgD were present as predicted from the results *in vitro*. More recently using a mouse monoclonal anti-idiotypic antibody raised against the surface IgM of patient ALL (Table I) (kindly provided by Dr M.G. Glennie) and a similar double-determinant ELISA assay, it has been possible to show that there is no idiotypic IgM in the serum of this patient. The molecular size of the serum idiotypic IgM was investigated for two of the patients (Car and Gol) by separation of 1 ml of serum on Ultrogel AcA 22 in the same way as for the culture fluids and was again shown to be pentameric.

Measurements of idiotypic IgM in the serum of patient Gol were made over a 38 week period during which treatment with two different drug combinations was carried out. Patient Gol, as described in Methods, presented with lymph node disease but later developed a leukaemia: the pattern of disease became one of increasing anaemia as tumour cells appeared in the bone marrow followed by a rapid increase in circulating tumour cells and clinical relapse. Such an episode is shown in Figure 1 where treatment with a drug combination of adriamycin, 6-thioguanine and cytosine arabinoside although producing an apparent clinical remission was followed ~10 weeks later by recurrent lymph node and spleen enlargement together with developing anaemia. By week 28 the patient had a high blood lymphocyte count and was given chlorambucil and prednisolone which brought about a dramatic response with a fall in circulating tumour cells and clinical improvement. The idiotype levels measured during this period are also

**Table III** Idiotypic Ig in patients' sera

| | Ig levels in serum[a] ($\mu g\,ml^{-1}$) | | | | | |
|---|---|---|---|---|---|---|
| *Patient* | *Total IgM* | *Idiotypic IgM* | *% Idiotype* | *Total IgD* | *Idiotypic IgD* | *% Idiotype* |
| Had | 390 | 24 | 6 | 10.2 | 0 | — |
| Car | 92 | 4 | 4 | 7.0 | 3.5 | 50 |
| Gol | 434 | 35 | 8 | 6.6 | 0 | — |
| Par | 185 | 4 | 2 | 6.3 | 0 | — |

[a]Serum samples were obtained from the patients ~2 months after cessation of chemotherapy (Had, Gol and Par) or radiotherapy (Car).

Ig levels were measured by the ELISIA technique. Anti-idiotypic antibody prepared for each patient was tested with normal human serum IgM and interference was ≤0.5%.

**Figure 1** Levels of idiotypic IgM in the serum of patient Gol during two treatments with chemotherapy. (O), blood lymphocyte count ($\times 10^{-9} l^{-1}$); vertical lines, idiotype IgM ($\mu g ml^{-1}$ serum). Treatment consisted of A, adriamycin, 6-thioguanine and cytosine arabinoside; B, chlorambucil and prednisolone.

indicated in Figure 1 where it can be seen that there was no fall during the first rather ineffective treatment and that levels were rising in the serum some weeks before any tumour cells could be seen. After the second treatment, idiotype levels fell dramatically as the patient improved clinically.

### Discussion

Patterns of secretion of Ig by unstimulated neoplastic B lymphocytes from patients with CLL have been studied previously and it has been shown that the majority (13/19 patients) do secrete small amounts of pentameric IgM which can be found in the patient's serum (Stevenson *et al.*, 1980). These low levels of secretion have been confirmed by others (Johnstone *et al.*, 1982) and the presence of intracellular IgM in cells from such patients has been detected by using sensitive immunoelectron microscopy (Yasuda *et al.*, 1982). Secretion of free monotypic light chain by the majority of CLL cell populations has also been observed (Hannam-Harris *et al.*, 1980). A smaller proportion of such cell populations (8/19) all of which had surface Ig of the λ light chain type, secreted monomeric IgD (Stevenson *et al.*, 1982).

Studies on the secretory capacity of neoplastic B lymphocytes have now been extended to patients with NHL for two main reasons: first, to investigate whether such cells have different patterns of secretion which might relate to the stage of differentiation at which arrest has occurred; and second, to examine secretory capacity in relation to production of soluble idiotypic Ig which would block an immunotherapeutic attack on the tumour cells by anti-idiotype. Conversely the serum idiotypic Ig should provide a useful tumour marker for monitoring disease levels.

Comparison of the data obtained on culturing monotypic lymph node cells from patients with NHL with that obtained previously from peripheral blood lymphocytes in CLL reveals that there are no major differences between the two diseases. Thus in CLL 19/19 secreted monotypic light chain and 13/19 secreted IgM (Stevenson *et al.*, 1982); in NHL 23/24 secreted light chain and 17/24 IgM. For IgD, in CLL 8/19 were positive whereas in NHL 6/21 were positive. Levels of secretion showed that CLL cells tended to secrete more free light chain ($1620 \pm 860$) than NHL ($1000 \pm 1050$) and less IgM ($170 \pm 93$) than NHL ($340 \pm 600$), all units being molecules $cell^{-1} h^{-1} \times 10^{-1}$, and given as mean $\pm$ SD but larger groups would have to be studied to assess the significance of the differences due to the heterogeneity in amounts secreted. A previous study of secretory patterns of cells from patients with NHL was carried out in this laboratory using the less direct technique of biosynthetic radiolabelling (Hannam-Harris *et al.*, 1982). Although it was more difficult to analyse the nature and amount of whole Ig secretion by this method, the measurement of light chain:heavy chain ratios showed a similar heterogeneous pattern.

Among the 24 cell populations from patients with NHL showing surface Ig of different classes and intensities there was no clear trend relating secretory capacity to surface Ig expression. Secretion of IgG was found in only three cell populations and further work is required to establish the clonal origin of this IgG.

The molecular size of the IgM in culture fluids was examined for three patients and was shown to be consistent with that of pentameric IgM. A similar finding was made for CLL cultures (Stevenson *et al.*, 1980) and indicates that the pathway of production is secretion rather than membrane "shedding". Surface labelling with lactoperoxidase was used previously to show that membrane Ig, if shed in the medium, is in a high molecular weight, probably vesicular form which does not enter the sizing column. The normal fate of surface IgM may well be via internalisation as has been suggested for other plasma membrane components (Doyle & Baumann, 1979).

The clonal origin of the secreted Ig has been demonstrated for four of the lymphoma patients for whom anti-idiotypic antibody was available. The idiotypic Ig was detectable in the serum of these patients although the percentage of the total serum IgM which was idiotypic (2–8%) was lower than that observed previously in CLL (57–93%), possibly reflecting different disease loads and complicated in these four patients by previous therapy. Correlation between the ability of cells to secrete idiotypic Ig *in vitro* and the measured level in serum cannot be examined without accurate assessment of disease load which is difficult to make. However, preliminary investigation of patient ALL whose cells secrete no Ig in culture has shown no idiotypic Ig to be present in the serum, making this patient a prime candiate for therapy with anti-idiotype. At the other end of the scale, the patient with a detectable IgM paraprotein in the serum had neoplastic cells which secreted IgM at a rate approximately forty times the average value for the 24 lymph node biopsies studied.

Idiotypic levels may be particularly useful for longitudinal studies on individual patients. The results on patient Gol showing a rise in serum idiotypic IgM occurring before any clear clinical changes followed by a fall post-chemotherapy indicate a good correlation with disease. As regards immunotherapy with anti-idiotype, which may have some promise in lymphoma (Hamblin *et al.*, 1980; Miller *et al.*, 1982), however, any circulating idiotype presents a problem which is only partly solved by plasmapheresis (Hamblin *et al.*, 1980). Culture of a small aliquot of a patient's tumour cells *in vitro* with measurement of secreted IgM should help predict suitable cases for treatment: this is now done as a routine in this laboratory before embarking on the raising of anti-idiotypic antibody.

We thank Alison Tutt for her invaluable assistance, Drs F. Macbeth and T.J. Hamblin for patient material and Professor D.H. Wright for histological data. This work was supported by Tenovus, Cardiff, the Leukaemia Research Fund and the Medical Research Council.

### References

DOYLE, D. & BAUMANN, H. (1979). Turnover of the plasma membrane of mammalian cells. *Life Sci.*, **24**, 951.

ENGVALL, E. & PERLMANN, P. (1972). Enzyme-linked immunosorbent assay, ELISA. III. Quantitation of specific antibodies by enzyme-labelled anti-immunoglobulin in antigen-coated tubes. *J. Immunol.*, **109**, 129.

GÉRARD-MARCHANT, R., HAMLIN, L., LENNERT, K., RILKE, F., STANSFELD, A.S. & VAN UNNICK, J.A.M. (1974). Classification of non-Hodgkin's lymphomas. *Lancet*, **ii**, 406.

HAMBLIN, T.J., ABDUL-AHAD, A.K., GORDON, J., STEVENSON, F.K. & STEVENSON, G.T. (1980). Preliminary experience in treating lymphocytic leukaemia with antibody to immunoglobulin idiotypes on the cell surfaces. *Br. J. Cancer*, **42**, 495.

HANNAM-HARRIS, A.C., GORDON, J. & SMITH, J.L. (1980). Immunoglobulin synthesis by neoplastic B lymphocytes: free light chain synthesis as a marker of B cell differentiation. *J. Immunol.*, **125**, 2177.

HANNAM-HARRIS, A.C., GORDON, J., WRIGHT, D.H. & SMITH, J.L. (1982). Correlation between Ig-synthesis patterns and lymphoma classification. *Br. J. Cancer,* **46,** 167.

JOHNSTONE, A.P., JENSENIUS, J.C., MILLARD, R.E. & HUDSON, L. (1982). Mitogen-stimulated immunoglobulin production by chronic lymphocytic leukaemic lymphocytes. *Clin. Exp. Immunol.,* **47,** 697.

LUKES, R.J. & COLLINS, R.D. (1974). Immunological characterization of human malignant lymphomas. *Cancer,* **34,** 1488.

MILLER, R.A., MALONEY, D.G., WARNKE, R. & LEVY, R. (1982). Treatment of B-cell lymphoma with monoclonal anti-idiotype antibody. *N. Engl. J. Med.,* **306,** 517.

NAIRN, R.C. (1976). *Fluorescent Protein Tracing,* 4th edn. Churchill Livingstone, Edinburgh. p. 369.

SALMON, S.E. & SELIGMANN, M. (1974). B cell neoplasia in man. *Lancet,* **ii,** 1230.

STEVENSON, F.K., HAMBLIN, T.J., STEVENSON, G.T. & TUTT, A.L. (1980). Extracellular idiotypic immunoglobulin arising from human leukemic lymphocytes. *J. Exp. Med.,* **152,** 1484.

STEVENSON, F.K., STEVENSON, G.T. & TUTT, A.L. (1982). The export of immunoglobulin D by human neoplastic B lymphocytes. *J. Exp. Med.,* **156,** 337.

STEVENSON, G.T., SMITH, J.L. & HAMBLIN, T.J. (1983). Immunological investigation of lymphoid neoplasms. In: *Practical Methods in Clinical Immunology,* (Ed. Nairn). Edinburgh: Churchill Livingstone, Edinburgh, Vol 6, p. 41.

TUTT, A.L., STEVENSON, F.K., SMITH, J.L. & STEVENSON, G.T. (1983) Antibodies against urinary light chain idiotypes as agents for detection and destruction of human neoplastic B lymphocytes. *J. Immunol.,* **131,** 3058.

YASUDA, N., KANOH, T., SHIRAKAWA, S. & UCHINO, H. (1982). Intracellular immunogobulin in lymphocytes from patients with chronic lymphocytic leukemia: an immunoelectron microscopic study. *Leuk. Res.,* **6,** 659.